# Utilizing Functional Genomics Screening to Identify Potentially Novel Drug Targets in Cancer Cell Spheroid Cultures

**DOI:** 10.3791/54738

**Published:** 2016-12-26

**Authors:** Eamonn Morrison, Patty Wai, Andri Leonidou, Philip Bland, Saira Khalique, Gillian Farnie, Frances Daley, Barrie Peck, Rachael Natrajan

**Affiliations:** ^1^The Breast Cancer Now Toby Robins Research Centre, Division of Breast Cancer, The Institute of Cancer Research; ^2^Division of Molecular Pathology, The Institute of Cancer Research; ^3^Institute of Cancer Sciences, University of Manchester

**Keywords:** Cancer Research, Issue 118, Functional, genomics, siRNA, spheroid, tumor, microenvironment

## Abstract

The identification of functional driver events in cancer is central to furthering our understanding of cancer biology and indispensable for the discovery of the next generation of novel drug targets. It is becoming apparent that more complex models of cancer are required to fully appreciate the contributing factors that drive tumorigenesis *in vivo* and increase the efficacy of novel therapies that make the transition from pre-clinical models to clinical trials.

Here we present a methodology for generating uniform and reproducible tumor spheroids that can be subjected to siRNA functional screening. These spheroids display many characteristics that are found in solid tumors that are not present in traditional two-dimension culture. We show that several commonly used breast cancer cell lines are amenable to this protocol. Furthermore, we provide proof-of-principle data utilizing the breast cancer cell line BT474, confirming their dependency on amplification of the epidermal growth factor receptor HER2 and mutation of phosphatidylinositol-4,5-biphosphate 3-kinase (PIK3CA) when grown as tumor spheroids. Finally, we are able to further investigate and confirm the spatial impact of these dependencies using immunohistochemistry.

**Figure:**
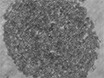


## Introduction

Solid tumors display significant histological, genetic and micro-environmental intra-tumor heterogeneity, which presents clinicians with a significant challenge in being able to treat patients successfully. The majority of models used to identify novel targeted therapies do not incorporate many of these features. Indeed, current targeted therapies utilized in the clinic have been developed in the last decade employing screening approaches that rely on cancer cell lines grown under two-dimensional (2D) culture conditions. Although this has brought about various successes, such as receptor tyrosine kinase inhibitors, it is becoming apparent that more complex models of cancer are required to fully appreciate the contributing factors that drive tumorigenesis* in vivo* and increase the number of new therapies that make the transition from pre-clinical models to clinical trials. Moreover, it is now well appreciated that 2D culture systems fail to reflect *in vivo* behavior ^1,2^. For example, in poorly vascularized tumors, as the demand for oxygen and nutrients within the microenvironment outstrip supply, regions of high and low deliveries develop. The presence of low oxygen (hypoxia) in tumors, as detected by the immunohistochemical staining of tumor sections for established hypoxic markers such as carbonic anhydrase IX (CAIX), correlates with a poorer clinical outcome in breast cancer ^3,4^ Thus incorporating features such as hypoxia into screening models may enhance our capacity to discover novel drug targets that will be more efficacious *in vivo*. Indeed, successfully targeting aggressive tumors that contain hypoxia is a clinical priority ^5^.

The altering of traditional 2D siRNA screening, in an attempt to more accurately recapitulate elements of the conditions encountered by cancer cells in the tumor microenvironment, has led to the identification of several genes that have been found to be important for tumor growth *in vivo*. These include functional genomic screens performed under low serum conditions ^6^, hypoxic conditions ^7^ and in combination ^8^. For example, silencing of 6-Phosphofructo-2-Kinase/Fructose-2,6-Biphosphatase 4 (PFKFB4), a protein responsible for regulating carbon entering glycolysis, only induced apoptosis in prostate cancer lines derived from metastasis when grown in low serum. Silencing of PFKFB4 in normal prostate cell lines under the same conditions had no effect, whereas, depletion of PFKFB4 completely ablated the growth of prostate cancer cell line xenografts ^6^.

In an extended panel of breast cancer cell lines, the silencing of mono-carboxylase transporter 4 (MCT4) preferentially led to the reduction of cell line growth under conditions of low oxygen. This vulnerability was validated *in vivo* in breast cancer cell line orthotropic xenografts. Perhaps most strikingly, silencing of Acetyl-CoA synthetase 2 (ACSS2), an enzyme responsible for converting acetate into acetyl-CoA, reduced cancer cell number under nutrient stress conditions (low oxygen and serum) but had little or no effect under normal culture conditions ^8^. Ablation of ACSS2 impacted the growth of breast and prostate cancer xenografts suggesting that nutrient gradients do not exist in isolation within the tumor microenvironment and that cells that reside in these regions are essential for tumor progression ^8^. Furthermore, ACSS2 was also found to be important in glioblastoma and hepatocellular carcinomas ^9,10^, suggesting that increased ACSS2 activity in tumors could be a fundamental mechanism that supports growth under unfavorable conditions.

Collectively, these studies prove that recapitulating the conditions encountered *in vivo* and performing siRNA screens allows for the identification of genes essential for cancer survival. As well as impacting 2D cancer cell growth under nutrient stress conditions, depletion of target genes in these studies inhibited cancer cell line spheroid growth, mirroring what was observed in tumor xenografts ^6,8^. Thus, cancer cell line spheroids contain several of the conditions encountered in the tumor microenvironment that impart sensitivity to ACSS2 silencing. Indeed, spheroids display nutrient gradients (Serum and oxygen), changes in pH, three-dimensional (3D) cell-cell contact, but also, alterations in proliferative celerity with cells undergoing cell cycle arrest and apoptosis. This is exemplified by the presence in cancer spheroids of necrotic regions, a feature not found in traditional 2D culture.

Cancer cell spheroids have already been used as more biologically relevant models to screen small molecule inhibitors but this only allows for the validation of compound efficacy or the repurposing of compounds originally design for other diseases ^11^. Current spheroid screening methods do not allow for the analysis of specific gene depletion in a high-throughput of high-content manner. Here, we describe for the first time, a functional genomics pipeline for uncovering specific gene dependencies utilizing small interfering RNA (siRNA) technology in cancer cell line spheroids. We designed a bespoke library with siRNAs targeting the 200 most frequently mutated genes in human breast cancers and assessed the impact of gene depletion on spheroid size and metabolic activity in BT474 breast cancer spheroids. We were able to robustly and reproducibly detect the impact of ERBB2 and PIK3CA silencing in 3D cultures. Moreover, we could assess the impact of gene depletion on the spatial architecture of BT474 spheroids using immunohistochemistry.

## Protocol

### 1. Preparation of 96-well siRNA Plates

NOTE: The outer edges of a 96-well plate are more prone to evaporation compared to other wells, thus limit the amount of siRNAs to 60 per 96-well plate. Fill in outer wells with plain medium or PBS to limit this. In addition, a validation screen of hits is recommended to alleviate plate biases.

1. Dilute the siRNAs to 250 - 500 ng/μL in reduced serum medium (as per manufacturer's recommendations) and aliquot 10 μL to the appropriate wells of the ultra-low attachment plate. Perform all screens in triplicate. NOTE: Incorporate appropriate non-targeting (negative) and killing (positive, such as UBB and PLK1) siRNA controls in the plate design to ensure transfection efficiency can be assessed. The use of low-attachment plates for the siRNA work is critical, as cells will be added to the transfection mixture. We cannot rule out that some manufacturers ultra-low attachment plates may have subtle effects on spheroid growth. As such we recommend that these are tested by the end user.
2. Cover plates with adhesive seals and store at -20 °C.

### 2. Reverse Transfection of Cell Line

1. On the day of the screen, defrost the plates at room temperature and spin for 5 min at 1,000 x g using a bench-top centrifuge. Add 10 μL of reduced serum medium containing the optimized-transfection reagent to each well using a multichannel pipette. Leave plates for 15 min for transfection complexes containing siRNA to form. NOTE: This study utilized the breast cancer cell line BT474 for functional genomic screening (cultured in high glucose DMEM (Dulbecco's Modified Eagle Medium) supplemented with 10% fetal bovine serum (FBS) and antibiotics).
2. Trypsinize the cells to be screened (0.05% trypsin and 0.53 mM EDTA) until they detach from the flask, neutralize using appropriate volume of cell line specific medium and spin for 5 min at 1,000 x g on a bench-top centrifuge to remove trypsin. Resuspend cells with an appropriate volume of medium and determine accurate cell number using a cell counter. NOTE: Typically 5,000 cells per well are sufficient for spheroid formation across most cell lines tested. It is important to accurately count the cells as a single cell suspension for accurate size comparisons.
3. Dilute cells to 5,000 cells per 180 μL in cold medium (4 °C). NOTE: The addition of reconstituted basement membrane matrix components will negatively impact transfection efficiency and it is recommended that it is not used. Cell line optimization to confirm spheroid forming capacity and knockdown efficacy should be performed prior to the screen.
4. Transfer the cell suspension to a reservoir, pipet to mix and add 180 µL to each well of the 96-well ultra-low attachment plate containing the siRNA prepared previously (2.1).
5. Centrifuge the plate at 1,000 x g in a precooled 4 °C centrifuge for 10 min and then return to a 37 °C tissue culture incubator.
6. NOTE: The cells will appear as a mosaic in the bottom of the wells. Over the next 12 - 24 h the cells will aggregate together to form a single sphere.
7. After 24 h, observe the cells form a single spheroid in the center of the well. Add 100 µL of complete medium to each well to encourage growth.
8. Replenish medium after three days. Gently remove 100 µL of medium from each well and add 100 µL of fresh medium.
9. On day 7, quantify the automated spheroid size on a plate reader that can quantitatively monitor spheroid growth over time (see Section 3).
10. Afterwards, determine cell viability using a luminescent cell viability dye (see Section 4).

### 3. Automated Image Acquisition

1. Scan the plates on a bench-top, micro-well plate imaging cytometer on day 7.
  1. Open the software, select the following: '96-well plate', select the appropriate plate type and enter an experiment name. NOTE: Any additional information can also be added into the software.
  2. Select the 'Tumorsphere' application. Alter the focus so that the spheroid is in focus and has optimal contrast. NOTE: We recommend 'image based focus' as significant variations in spheroid sizes are expected.
  3. Select the wells that require scanning and 'Start scan'.
  4. Using the plate scanner software, ensure that the object mask accurately represents the spheroid size. Do this by adjusting the colony diameter, border dilation, minimum thickness and precision settings, specific to each cell line tested ^11,12^. NOTE: The spheroid area will then be calculated using the software algorithm. For example, to accurately calculate the area of BT474 spheroids grown for seven days adjust precision to 'high' and set the minimum colony diameter to 200 µM. This should produce an appropriate spheroid mask representative of the spheroid area. NOTE: These data can then be exported from the machine, using the export function, in in the form of an annotated data file. Date is plate median normalized, combined and analyzed either by z-score or strictly standardized mean difference (SSMD) to identify siRNAs that had a statistically significant effect on spheroid area.

### 4. Determining Cell Viability

1. After spheroid area has been calculated, determine the viability using a luminescent viability cell dye. Prepare the reagent as per the manufacturer's instructions.
2. Carefully remove 100 µL of medium from each well and add 100 µL of viability dye. Incubate plates for 15 min then scan using a luminescent plate reader. NOTE: These data can then be exported from the machine, using the export function, in in the form of an annotated data file. Date is plate median normalized, combined and analyzed either by z-score or strictly standardized mean difference (SSMD) to identify siRNAs that had a statistically significant effect on spheroid viability.

## Representative Results

Spheroid assays in ultra-low attachment 96-well plates provide a high-throughput phenotypic assessment for potential oncogenicity in a context that more readily recapitulates the physiological conditions found in tumors *in vivo*. Indeed, the cancer cell lines MCF10DCIS.com and BT474 form tight spheroid structures (**Figure 1A**) and immunohistological investigation of spheroid sections showed distinct spatial changes in cellular and nuclear morphology. Over time, some spheroids such as BT474 spheroids develop necrotic regions, a common feature of aggressive solid tumors (**Figure 1B**). Some spheroids do not develop necrotic cores, such as the MDA-MB-231 cell line, but do display marked variation in the proliferative marker Ki67, which inversely correlates with cleaved caspsase-3 expression, a marker of apoptosis (**Figure 1C**). To establish that multiple cell lines are indeed receptive to siRNA-mediated gene silencing BT474, MCF10DCIS.com, MDA-MB-231 and JIMT1 cells were reverse transfected with siRNA for seven days. The presence of transfection reagent (mock) or transfection of control siRNAs had no effect on spheroid viability, while silencing of the essential gene Ubiquitin B (UBB) significantly reduced spheroid viability in all the cell lines tested (**Figure 1D**).

We designed a human siRNA library that encompassed the most frequently mutated genes in unselected, ER+, HER2+ and triple negative breast cancers. This library consists of genes of known function, such as MYC, PIK3CA and TP53 and those whose contribution to carcinogenesis is not established. The screen also contains several non-targeting control siRNAs (Control #1, Control #2) and siRNAs targeting essential genes, such as PLK1 and UBB, which act as killing controls (**Table 1**). We chose to use the breast cancer cell lines BT474 as they readily form spheroids without the addition of reconstituted basement membrane, are established workhorse cell lines and have a known genomic architecture. For example, BT474 cells are positive for the estrogen receptor (ER+), overexpress the human epidermal growth factor receptor 2 (HER2+) and harbor mutations in TP53 (E285K) and PIK3CA (K111N)^13^.

Employing the protocol outlined above, we monitored the impact gene depletion had on spheroid size and viability after seven days of siRNA reverse transfection (**Figure 1E**). Interestingly, the majority of genes did not have a significant effect on spheroid area or viability (**Figure 2A**). Silencing of FOXO3, PIK3CA, ERBB2 and SF3B1 resulted in the most significant reproducible reduction in spheroid size. This reduction was also observed in spheroid viability after ERBB2 and SF3B1 silencing. Encouragingly, we confirmed the impact of PIK3CA, ERBB2 and SF3B1 silencing on spheroid size using bright-field microscopy (**Figure 2B**). We previously identified SF3B1 as an essential gene in numerous cell line models and thus siSF3B1 represents a good killing control in addition to UBB ^14^. Interestingly, of all 200 genes only the silencing of E-Cadherin resulted in a significant increase in spheroid viability (**Figure 2A**). Investigation of spheroid morphology showed that E-Cadherin silencing resulted in a complete breakdown of spheroid architecture, with viable cells resting on the bottom of the low attachment well (**Figure 2B**). Manual reinvestigation of the spheroid volume screen data showed that this had also been observed but had been eliminated from area quantification due to the object being above the set size restrictions. As previously highlighted, BT474 cells overexpress the receptor tyrosine kinase HER2 and harbor an oncogenic mutation in PIK3CA (K111N). We confirmed that silencing of ERBB2 and PIK3CA resulted in reduced spheroid viability, while transfection with non-targeting controls had no effect (**Figure 2C**).

Next we investigated the impact of siRNA depletion on spheroid histology. BT474 spheroids were reverse transfected with non-targeting control siRNA and siRNAs targeting PIK3CA, ERBB2 and UBB. Silencing of ERBB2 and UBB resulted in a reduction of the pro-proliferative marker Ki67 compared to control siRNA (**Figure 2D**). The activation of the pro-apoptotic marker cleaved caspase-3 was only observed after UBB silencing, suggesting that depletion of HER2 and PIK3CA did not result in apoptosis but were cytostatic rather than cytotoxic. Indeed, silencing of HER2 and PIK3CA did result in an increase in the protein expression of the cell cycle arrest protein p27 compared to control transfected spheroids.

Taken together, these results show that BT474 cells are driven by oncogenic HER2 and PIK3CA signaling when grown as 3D spheroids. More importantly, these results show that it is possible to design and implement a bespoke siRNA screening library of hundreds of genes in cancer cell line spheroids robustly and reproducibly.


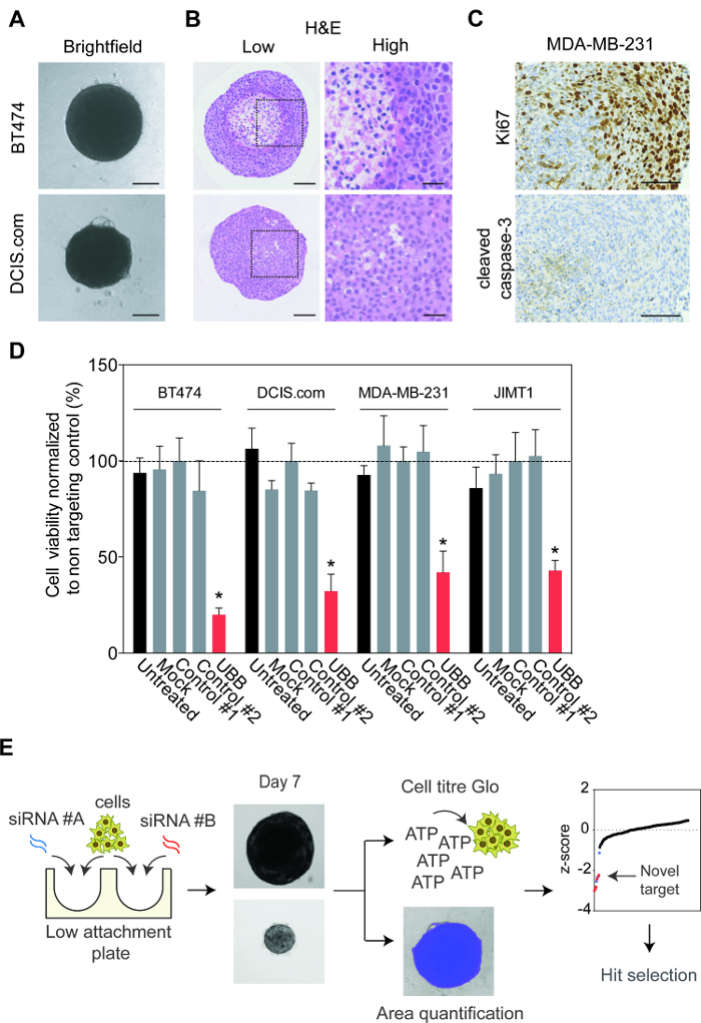
**Figure 1:Cell Line Optimization for 3-dimensional Growth.A.** The breast cancer cell lines, MCF10DCIS.com and BT474 were cultured in low attachment plates for 7 days. Brightfield representative images were taken using an inverted microscope. Scale bars = 100 µm. **B.** MCF10DCIS.com and BT474 spheroids were cultured for 28 days. 100 μL of fresh media was replenished every 3 - 4 days. Spheroids were fixed in 3.8% formaldehyde, embedded, sectioned and stained with hematoxylin and eosin (H&E). Representative images are shown at low and high magnification. Scale bars represent 100 µm and 33 µm, respectively. **C.** MDA-MB-231 spheroids were cultured for 21 days. 100 μL of fresh media was replenished every 3 - 4 days. Spheroids were fixed in 3.8% formaldehyde, embedded, sectioned and stained with Ki67 and cleaved caspase-3. Representative images are shown. Scale bars = 100 µm. **D.** BT474, MCF10DCIS.com, MDA-MB-231, and JIMT1 cell lines were reverse transfected with mock (transfection reagent only) and the control siRNAs and UBB, ultra-low attachment plates were then spun to form spheroids. Fresh medium (100 µl) was added on days 1 and 4. After 7 days, cell viability was quantified. Data represent the mean ± SD of two independent biological replicates performed in triplicate normalized to control #1. Statistical significance was calculated using an unpaired Students t-test (*p*< 0.05). **E.** A flow diagram summarizing the reverse transfection protocol used to functionally interrogate gene dependency in cancer cell line spheroids. Please click here to view a larger version of this figure.


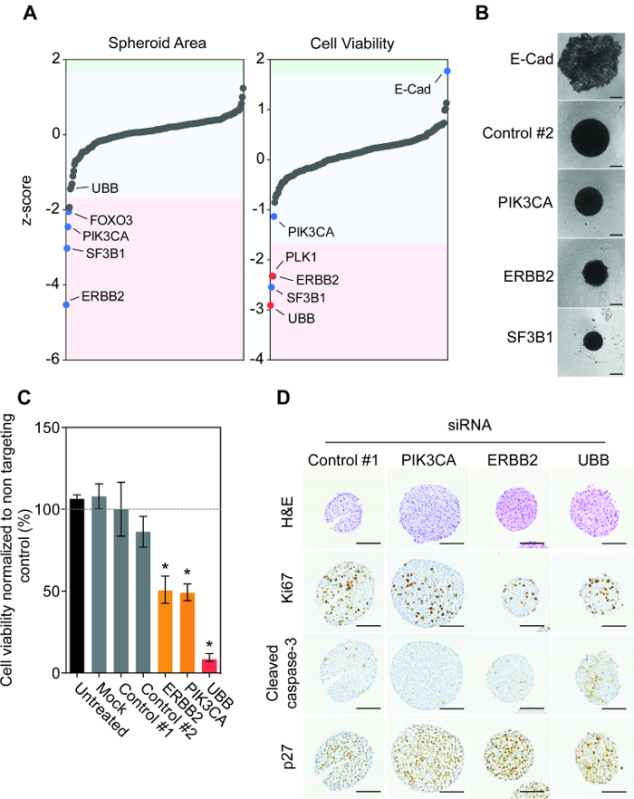
**Figure 2:Functional Genomic Investigation of BT474 Spheroids Uncovers Oncogenic Dependencies. A.** BT474 cells were reverse transfected with the 200-gene human siGENOME siRNA library in triplicate. Spheroid size and viability was observed. Raw data values were plate median normalized and z-scores were calculated to identify significant outliers greater than 1.7x the standard deviation of the plate median ^15^. Note outlying genes ERBB2, SF3B1, PLK1 and UBB and E-cad. siRNAs that significantly increased or reduced spheroid area and viability are shaded in blue and red, where red depicts the control siRNA's. **B.** Cells were reverse transfected with non-targeting control, E-Cadherin (E-Cad), PIK3CA, ERBB2 or UBB siRNA and then spun in an ultra-low attachment plate to form spheroids. After 7 days, brightfield representative spheroid images were taken using an inverted microscope. Scale bars = 100 µm. **C.** Cells were reverse transfected with non-targeting control, PIK3CA, ERBB2 or UBB siRNA and then spun in an ultra-low attachment plate to form spheroids. After 7 days, cell viability was quantified. Data represent the mean ± SD of two independent biological replicates performed in triplicate normalized to control #1. Statistical significance was calculated using an unpaired Students t-test (*p *< 0.05). **D.** After 7 days, spheroids were fixed, embedded, sectioned and stained for H&E, Ki67, Cleaved Caspase-3, and p27. Representative images are shown. Scale bars = 100 µm. Note the H&E of the siControl spheroids appear smaller due to an artefact of processing and individual intact spheroids chosen for staining. However this does not detract from the changes observed with the scanned images and cell viability results. Please click here to view a larger version of this figure.

**Table:** 

1	2	3	4	5	6	7	8	9	10	11	12
											
	Non-targeting 1	TP53	DST	KMT2C	Untreated 1	FCGBP	ARID1B	FBXM7	TTC40	Non-targeting 2	
	GATA3	TTN	MUC12	MUC4	AHNAK	HUWEI1	DNAH11	ITPR2	ABCA13	CREBBP	
	MAP2K4	PIK3CA	F5	APOB	ANKRD30A	MUC17	DNAH17	LAMA2	ACE	CSMD2	
	STARD9	USH2A	FAT3	LPR2	CSMD3	MYO18B	DNAH5	MDN1	ARHGAP5	DNAH9	
	CXCR3	MUC16	RB1	PKHD1L1	DNAH2	SYNE2	DYNC1H1	PCLO	CACNA1B	ERBB2	
	PLK1	SYNE1	LYST	PTEN	SPTA1	Untreated 2	VHL	RYR1	COL6A3	UBB	
											
1	2	3	4	5	6	7	8	9	10	11	12
											
	Non-targeting 1	ENAM	RYR2	BRCA2	Untreated 1	FMN2	HECW1	LAMB4	SI	Non-targeting 2	
	FHOD3	MACF1	RYR3	C2ORF16	DMD	FRG1	HERC2	MYH11	STAB1	ZDBF2	
	GOLGA6L2	NEB	SMG1	CACNA1E	DNA14	GCC2	HIVEP2	NIPBL	TANC1	ZNF536	
	HMCN1	NF1	UBR5	CACNA1F	DYNC2H1	GON4L	HYDIN	PKD1L1	TF	ANK3	
	HRNR	OBSCN	USP34	CYMA5	FAM208B	GPR112	ITSN2	RNF213	TPR	ASPM	
	PLK1	PCDH15	XIRP2	COL7A1	FLG2	Untreated 2	VHL	SAGE1	UNC80	UBB	
											
1	2	3	4	5	6	7	8	9	10	11	12
											
	Non-targeting 1	DCHS2	MUC5B	ZFHX4	Untreated 1	MYO9A	SPHKAP	CXORF22	NCOR1	VPS13D	
	ATR	DMXL2	MXRA5	ANK2	KIAA1210	PRUNE2	TCHH	DANH6	NOTCH2	ANKRD12	
	BIRC6	DNAH10	TENM1	ATM	LRP1	SCN10A	VPS13C	DNAH7	SPEN	C5ORF42	
	CDH1	DNAH3	PEG3	DIDO1	MAP1A	SCN2A	IPTR3	ERBB3	SRRM2	CCDC88A	
	CUBN	NOCK11	RELN	DNAH8	MED12	SHROOM2	CEP350	FAT4	SZT2	CHD4	
	PLK1	EYS	SACS	KIAA1109	MED13	Untreated 2	VHL	KMT2A	VPS13A	UBB	
											
1	2	3	4	5	6	7	8	9	10	11	12
											
	Non-targeting	QSER1	ARID1A	WDFY3	Untreated 1	SDK1	TEX15	LAMA1		Non-targeting 2	
	COL14A1	SHROOM3	ATRX	EFCAB5	SF3B1	CBFB	AHNAK2				
	CSMD1	TBX3	KIAA0947	FOXA1	ITPR1	DDX3X	KIF4A				
	MEFV	UBR4	MYCBP2	INPPL1	FLG	HECTD4	FAT2				
	MGAM	VCAN	NBEAL1	MAP3K1	AKAP9	GPR98	FOXO3				
	PLK1	ZNF462	SETX	NRP1	HERC1	Untreated 2	VHL			UBB	
											

**Table 1: Plate Layout and Human siRNA Library De-convolution. **The table contains the content of each of the siRNA pools and layout for the low attachment plates used in the screen.

## Discussion

Three-dimensional models of cancer are being increasingly employed to evaluate the efficacy of known and novel compounds that have been designed to selectively kill cancer cells. Cancer cell spheroids are structures that display conditions more similar to those encountered in tumors *in vivo, *thus compounds that have increased efficacy in 3D are more likely to have an effect *in vivo. *However, these modalities do not allow for the identification of potentially novel targets that have not been the subject of drug design that could have considerable efficacy in treating cancer.

We developed a siRNA functional genomics approach that allowed for durable gene silencing for up to seven days in cancer cell line spheroids. There are several critical steps to the protocol that require optimization before a siRNA screen can be performed. The ability to form reproducible viable spheroids on a large scale is essential. Moreover, appropriate transfection conditions should be rigorously optimized. We suggest trialing several different transfection reagents with appropriate non-targeting and killing controls prior to attempting the screen. We were able to show that several commonly used breast cancer cell lines, namely BT474, MCF10DCIS.com, MDA-MB-231 and JIMT1 were amenable to siRNA transfection. Furthermore, we provide proof-of-principle data screening the 200 most frequently mutated genes in breast cancer in BT474 spheroids, confirmed their dependency on amplification of HER2 and oncogenic mutation of PIK3CA. Interestingly, silencing of the transcription factor FOXO3 resulted in a reduction in spheroid size but no significant effect on viability. FOXO3 is known to regulate the response to hypoxia, altering the metabolic capacity of cancer cells allowing them to adapt more readily to their environment ^16^. This role could potentially interfere with the cell viability reading as it detects ATP abundance, one of the main products of cell metabolism.

In support of the observation of a reduction in spheroid size, it has been shown that silencing of FOXO3 in HeLa xenografts impaired tumor growth and induced apoptosis ^17^. It is important to note that some genes may impact the capacity of cancer cells to retain their 3D architecture, which could result in false positives. For example, knockdown of E-Cadherin resulted in dissolution of BT474 spheroid structure. This had been previously reported using E-Cadherin targeted antibodies ^18^. As with any screening platform, potential targets should be rescreened to assess the reproducibility of the effect observed. There are limitations to the technique, namely the transient nature of the siRNA-mediated gene knockdown. Sustained silencing longer than seven days was not achievable with siRNA.

The advantage of this approach is that it can be coupled with various other biometric dyes not just those that assess spheroid viability, for example, giving spatial information of spheroid hypoxia or monitoring cells undergoing apoptosis. Moreover, because the plate reader scans are relatively quick and non-invasive, the impact of siRNAs on spheroid size can be assessed over time rather than just at the experimental end point. Indeed, we are currently exploring several of these avenues within our screening pipeline. An alternative approach that utilizes 3D cultures to identify novel dependencies is the use of chemical libraries that inhibit either a broad range of targets or particular families of proteins. Indeed, Bitler *et al*. utilized this targeted approach to identify the synthetic lethality interaction between ARID1A status and EZH2 inhibitors in ovarian clear cell carcinomas ^19^. The discovery of CRISPR-Cas9 gene editing technology has also allowed for the development of genetic screens in organoid cultures and *in vivo. *However, this approach is dependent on having appropriate animal facilities and may be cost prohibitive ^20^.

In conclusion, we believe that we have outlined a protocol that more accurately models the oxygen and nutrient gradients, which are features of the tumor microenvironment *in vivo*, allowing for the identification of novel cancer targets or robust validation of established targets. Moreover, our protocol can be applied to any type of cell line that forms spheroids and hence can be routinely used in the cancer research community for high-throughput siRNA screens.

## Disclosures

Open Access fees were supported by Nexcelom Bioscience, LLC.
